# Population genomics of the Asian tiger mosquito, *Aedes albopictus*: insights into the recent worldwide invasion

**DOI:** 10.1002/ece3.3514

**Published:** 2017-10-24

**Authors:** Panayiota Kotsakiozi, Joshua B. Richardson, Verena Pichler, Guido Favia, Ademir J. Martins, Sandra Urbanelli, Peter A. Armbruster, Adalgisa Caccone

**Affiliations:** ^1^ Department of Ecology and Evolutionary Biology Yale UniversityNew Haven CT USA; ^2^ Department of Public Health and Infectious Disease Sapienza University of Rome Rome Italy; ^3^ School of Bioscience and Veterinary Medicine University of Camerino Camerino Italy; ^4^ Laboratório de Fisiologia e Controle de Artrópodes Vetores IOC‐FIOCRUZ Rio de Janeiro Brazil; ^5^ Department of Environmental Biology Sapienza University of Rome Rome Italy; ^6^ Department of Biology Georgetown University Washington DC USA

**Keywords:** arboviruses vector, ddRAD, genetic structure, phylogeography, SNPs

## Abstract

*Aedes albopictus*, the “Asian tiger mosquito,” is an aggressive biting mosquito native to Asia that has colonized all continents except Antarctica during the last ~30–40 years. The species is of great public health concern as it can transmit at least 26 arboviruses, including dengue, chikungunya, and Zika viruses. In this study, using double‐digest Restriction site‐Associated DNA (ddRAD) sequencing, we developed a panel of ~58,000 single nucleotide polymorphisms (SNPs) based on 20 worldwide *Ae. albopictus* populations representing both the invasive and the native range. We used this genomic‐based approach to study the genetic structure and the differentiation of *Ae. albopictus* populations and to understand origin(s) and dynamics of the recent invasions. Our analyses indicated the existence of two major genetically differentiated population clusters, each one including both native and invasive populations. The detection of additional genetic structure within each major cluster supports that these SNPs can detect differentiation at a global and local scale, while the similar levels of genomic diversity between native and invasive range populations support the scenario of multiple invasions or colonization by a large number of propagules. Finally, our results revealed the possible source(s) of the recent invasion in Americas, Europe, and Africa, a finding with important implications for vector‐control strategies.

## 1 INTRODUCTION

**Figure 1:**
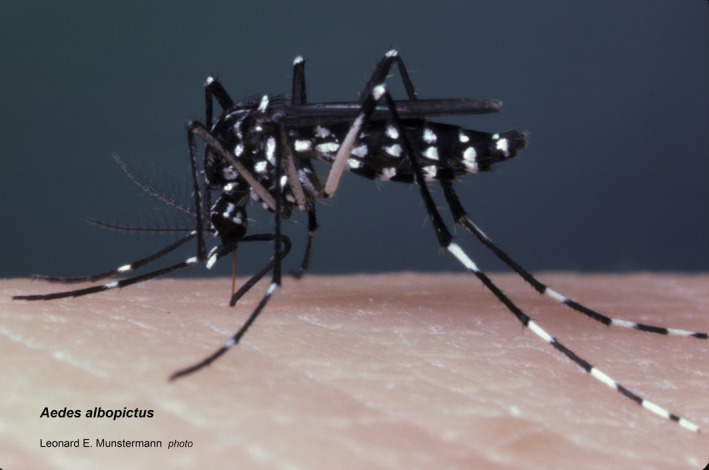
The Asian tiger mosquito, *Aedes albopictus*. *Photograph Credit: Leonard Munstermann*

The Asian tiger mosquito, *Aedes albopictus* (Figure 1), is one of the 100 most successful invasive species in the world (Bonizzoni, Gasperi, Chen, & James, 2013; Hawley, 1988; Invasive Species Specialist group 2017) as it has invaded Europe, Western Africa, and Southern Africa as well as North and South America during the last 30–40 years (Medlock, Hansford, Schaffner, Versteirt, Hendrickx, Zeller, & Bortel, 2012; Paupy, Delatte, Bagny, Corbel, & Fontenille, 2009; Reiter & Darsie, 1984; Sprenger & Wuithiranyagool, 1986). The species is native to the Oriental region, where it is distributed throughout Southeast Asia, China, and Japan (Bonizzoni et al., 2013; Hawley, 1988) but it has also colonized southwest Indian Ocean islands as early as ~1500 years BP (Goubert, Minard, Vieira, & Boulesteix, 2016), and Pacific islands including Hawaii and Guam likely over 100 years ago (Lounibos, 2002). *Ae. albopictus* is a significant biting pest and of public health concern (Medlock et al., 2012) because it is a competent vector of at least 26 arboviruses, including dengue (DENV), chikungunya (CHIKV), and Zika (ZIKV) viruses (Benedict, Levine, Hawley, & Lounibos, 2007; Gratz, 2004; Liu et al., 2017; Smartt et al., 2017; Wong, Li, Chong, Ng, & Tan, 2013). Its role as a primary vector of agents of recent outbreaks of both dengue fever (caused by DENV) and chikungunya fever (caused by CHIKV) (Bonizzoni, Gasperi, Chen, & James, 2013; Morens & Fauci, 2008; Paupy, Delatte, Bagny, Corbel, & Fontenille, 2009; Wu, Lun, James, & Chen, 2010) is well established. Although its competence for the DENV and ZIKV virus does not seem to be as high as *Ae. aegypti* (Brady, Golding, Pigott, Kraemer, Messina, Reiner Jr, ..., Hay, 2014; Chouin‐Carneiro, Vega‐Rua, Vazeille, Yebakima, Girod, Goindin, ..., Failloux, 2016; de Lamballerie et al., 2008), its ability to invade and persist in temperate areas causes serious concerns for its potential role in transmission of these and other viruses (Liu et al., 2017; Smartt et al., 2017; Wong, Li, Chong, Ng, & Tan 2013).

Despite its epidemiological importance, detailed information on the evolutionary history of the worldwide range expansion of *Ae*. *albopictus* is lacking. Many of the phylogeographic and the population genetic studies conducted using nuclear and mitochondrial (mtDNA) markers provided limited resolution because of a combination of factors, including low levels of variation in certain mtDNA markers [but see (Ismail et al., 2015; Porretta, Mastrantonio, Bellini, Somboon, & Urbanelli, 2012; Zhong et al., 2013)], limited population sampling from across the range of the species, and the inability to combine datasets from different studies [for a review on markers used see (Goubert, Minard, Vieira, & Boulesteix, 2016)]. Recent studies utilizing highly variable microsatellite markers represent a significant advance and have begun to illuminate processes of both regional (Maynard et al., 2017; Medley, Jenkins, & Hoffman, 2015) and global range expansion (Manni et al., 2017). Although the previous studies provided important insights into the possible origin of the invasions, their results were sometimes contradictory [e.g., the case of Greece (Kamgang et al., 2011; Manni et al., 2017) or Brazil (Birungi & Munstermann, 2002; Kambhampati, Black, & Rai, 1991)]. However, determining the origin of the invasions unequivocally and/or at a high level of resolution would be valuable for a variety of public health interventions (Beebe et al., 2013; Delatte et al., 2013; Galtier, Nabholz, Glemin, & Hurst, 2009; Goubert, Minard, Vieira, & Boulesteix, 2016; Hurst & Jiggins, 2005; Manni et al., 2015; Medley, Jenkins, & Hoffman, 2015; Mousson et al., 2005; Porretta, Gargani, Bellini, Calvitti, & Urbanelli, 2006; Zhong et al., 2013). First, knowledge regarding the source of an introduction's origin(s) can provide information on the invasive population's likely mode of transportation (Goubert et al., 2016; Jackson et al., 2015; Powell & Tabachnick, 2013). Similarly, because different insecticides are used in different parts of the world, identifying invasion sources can help provide information on the likelihood of insecticide resistance in newly invasive populations (Hemingway & Ranson, 2000). Finally, because *Ae. albopictus* populations vary in viral competence (Chouin‐Carneiro et al., 2016; Lambrechts et al., 2009), understanding whether an introduction is from an active transmission region will help to assess the public health threat of the invasion. Single nucleotide polymorphisms (SNPs) are extremely powerful genetic markers that are densely distributed across eukaryotic genomes and provide a basis for high‐resolution analysis of historical biogeography and invasion dynamics (Emerson et al., 2010; Puckett et al., 2016).

Genomewide SNPs can also provide a valuable tool for identifying the genetic basis of important ecological adaptations, including traits related to invasion success and range expansion (Wray, 2013). Such information may provide the basis of novel vector‐control strategies based on the genetic or chemical disruption of these adaptations. In the case of *Ae. albopictus*, two life‐history traits are particularly important to ecological adaptations during the range expansion of this species. First, its affinity to human‐made containers and environments allowed this species to quickly expand its range within and among continents due to regional and global trade among distant geographic regions (Medley, Jenkins, & Hoffman, 2015; Tatem, Hay, & Rogers, 2006) as has been observed in other *Aedes* species as well (Damal, Murrell, Juliano, Conn, & Loew, 2013; Egizi, Kiser, Abadam, & Fonseca, 2016). Second, the capacity for facultative photoperiodic diapause (Hawley, 1988; Mori, Oda, & Wada, 1981; Urbanski, Benoit, Michaud, Denlinger, & Armbruster, 2010) is largely responsible for the capacity of this mosquito to adapt to a temperate climate, enabling its range expansion into regions at higher latitudes in North America and North Europe (Armbruster, 2016; Becker et al., 2013; Flacio, Engeler, Tonolla, & Müller, 2016; Urbanski et al., 2010). Diapause is a preprogrammed, hormonally controlled dormancy that enables many insects to survive the unfavorable conditions of temperate winters (Denlinger & Armbruster, 2014).

Here, we use a population genomic approach to fill these knowledge gaps. We used the ddRAD‐seq method (Peterson, Weber, Kay, & Fisher, 2012) to obtain a densely distributed set of genomewide marker SNPs. We screen for SNP variation within and among 20 *Ae. albopictus* populations worldwide from both the native and the invasive range. The goals of this work are to (1) study the genetic structure of *Ae. albopictus* populations worldwide, (2) identify the possible source(s) of the recent invasions in Europe, the Americas and Africa, (3) estimate the genetic diversity and differentiation between *Ae. albopictus* populations and compare between the invasive and the native range, and (4) provide a pool of SNPs as a baseline for future population genomic and genetic mapping studies.

## 2 METHODS

### 2.1 Mosquito collections, DNA extraction, and ddRAD‐seq libraries preparation

*Aedes albopictus* field samples included adults or eggs. Adults were preserved in 70%–100% ethanol or dry at −80°C until DNA extraction. Eggs were collected from multiple ovitraps to avoid sampling siblings and then hatched in the laboratory. Adults or larvae were then stored as above. This study includes four to six mosquitoes/locality from 20 localities worldwide (Table 1). We used a small number of individuals per locality because, small sample sizes [as for example used in (Brown et al., 2014; Puckett et al., 2016; Trucchi et al., 2016; Willing et al., 2010)] can be highly informative for studying the genetic differentiation and the evolutionary relationships of populations when screening tens of thousands of markers (Nazareno, Bemmels, Dick, & Lohmann, 2017; Patterson, Price, & Reich, 2006). Specifically, according to Nazareno, Bemmels, Dick, & Lohmann, (2017) even two samples per population are adequate when >1,500 SNPs are used and according to the estimations of Patterson, Price, & Reich (2006), if the true Fst between two populations is 0.01 using ~1,000 SNPs, one will need 10 individuals/population. Thus, given the use of >50K SNPs and our Fst estimates (see below in the Results section) and the ones from microsatellites studies (Beebe et al., 2013; Das, Satapathy, Kar, & Hazra, 2014; Kamgang et al., 2011; Manni et al., 2015, 2017; Maynard et al., 2017; Minard et al., 2015; Pech‐May et al., 2016), sample size used here is well within what is considered adequate. However, to ensure that this is valid in our case organism, we performed some preliminary analyses using two populations (Greece and Italy) of 11 and 16 samples and subsequently, we reduced the number of samples to four for each one and repeated the analyses. Our results confirmed that for the specific analyses performed in this study (population genetics analyses and phylogeographic analysis), the sample size used even though small, it is adequate (results provided in Appendix).

**Table 1:** Population information for the *Aedes albopictus* samples used in this study

Locality [map code]	Country	Range	Year	*N*	Gen lab	Code
Itacoatiara, Amazon State [01]	Brazil	Invasive	2015	4	F0	COAT
Presidente Figueiredo, Amazon State [02]	Brazil	Invasive	2015	4	F0	PRES
Salvador [03]	Brazil	Invasive	2001	6	F3	SALV
Kinshasa [04]	DRC	Invasive	2011	4	F0	DRC
Franceville [05]	Gabon	Invasive	2015	4	F2	FCV
Greece [06]	Greece	Invasive	2013	4	F0	GRE
San Benedetto del Tronto [07]	Italy	Invasive	2008	36	F35	ITA‐COL
Rome [08]	Italy	Invasive	2005	4^a^	F0	ITA‐ROM
Brownsville, Texas [09]	USA	Invasive	2010	4	F0	BRO
Corpus Christi, Texas [10]	USA	Invasive	2001	4	F0	CORP
Florida [11]	USA	Invasive	2006	6	F1	FLO
Hawaii [12]	USA	Invasive	2006	4	F3	HAW
Newark, New Jersey [13]	USA	Invasive	2008	4	F0	NEW
Manassas, Virginia [14]	USA	Invasive	2010	4	F0	VIRG
Bermuda [15]	BT	Invasive	2015	4	F0	BER
Kagoshima [16]	Japan	Native	2008	4	F0	KAG
Tokyo [17]	Japan	Native	2006	6	F0	TOK
Kuala Lampur [18]	Malaysia	Native	2006	6^a^	F3	KLP
Sentosa Island [19]	Singapore	Native	2014	4	F0	SEN
Phu Hoa [20]	Vietnam	Native	2015	4	F12	VIET

**Figure 2:**
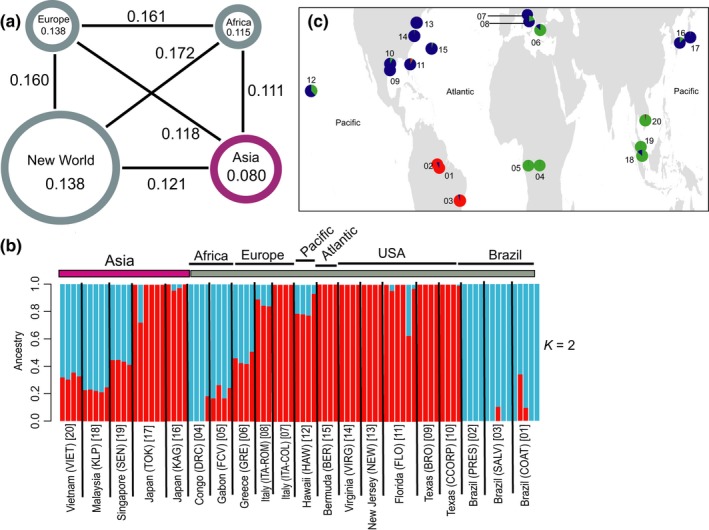
Genetic structure and differentiation of the populations used in the study. (a) Average pairwise Fst values between (indicated by black lines connecting each pair) and within continents (indicated in the circles). The size of the circles is proportional to the number of populations sampled, and colors represent the native (purple) and the invasive (gray) range. (b) ADMIXTURE barplot (*K* = 2, best supported grouping) for all *Aedes albopictus* populations. Individuals are vertical bars along the plot. The *Y* axis represents the percentage of each individual (*Q* value) assigned to a cluster; the height of each color represents the probability of assignment to a genetic cluster. The black vertical lines indicate population limits. The bars above the plot indicate the native (purple) and the invasive (gray) species range. Population names are reported on the *X* axis. (c) Pie charts representing the mean Admixture *Q* values for three groups (*K* = 3) clustering as indicated by the Admixture analysis for each *Ae. albopictus* sampling locality. Population code numbers in brackets as in Table1

Year, year of collection; *N*, number of individuals used in the study; gen lab, Number of generations reared in laboratory conditions; Code, population code used for the downstream analyses; DRC, Democratic Republic of Congo; BT, British Overseas Territory. Map codes refer to labels in Figure 2C and population code are consistent in all the figures of the study.

a One individual mosquito excluded from subsequent analyses because of poor sequencing quality.

DNA was extracted using the DNeasy Blood and Tissue kit (Qiagen), according to the manufacturer's instructions but with an additional RNAse A (Qiagen) step. Double‐digest restriction site‐associated DNA (ddRAD) sequencing libraries were prepared according to Peterson , Weber, Kay, & Fisher (2012), as modified by Gloria‐Soria et al. (2016). Briefly, for the ddRAD library preparation, ~500–700 ng of high‐quality DNA was simultaneously doubled‐digested using NlaIII and MluCI (NEB) restriction enzymes (REs) following manufacturer's instructions. The individual bar coding was followed by polymerase chain reaction (PCR) amplification (eight cycles). We then pooled 16 bar‐coded samples in each library and proceeded to size selection, using the Blue Pippin electrophoresis platform (Sage Science). We selected fragments of 215 bp (base pair) under the “tight” setting. Libraries were sequenced (75‐bp paired‐read sequencing), using the Illumina Hi‐Seq 2000 platform at the Yale Center for Genome Analysis. To achieve the best sequence quality, the complexity of the sequencing lanes was increased by spiking the libraries with another library constructed using different REs.

### 2.2 Sequence Data processing

Sequence data (reads) were de‐multiplexed and mapped against the *Ae. albopictus* reference genome (Chen et al., 2015) using Bowtie2 v.2.1.0 (Langmead & Salzberg, 2012) and Samtools v. 1.3 (Li et al., 2009). Variant calling was performed using Sam tools based on the full dataset of all the populations distributed worldwide. The variant filtering carried out using the VCFtools v. 0.1.14.10 (Danecek et al., 2011) and the following parameters: The reads that aligned to the reference genome with a minimum mapping quality of Q10 were retained, and then, only biallelic SNPs with genotype depth (minDP) >5.0X were included in our dataset. Then, we created three datasets: (1) global, (2) invasive, and (3) native based on the distribution of the populations (Table 1). Subsequently, each dataset was further filtered to retain SNPs with a minor allele frequency (MAF) > 0.05 and genotyped in at least 70% of the samples. We also used Q20 as minimum mapping quality to explore the impact of varying this parameter. As expected, this preliminary dataset resulted in a much lower number of SNPs than the one using Q10, but conclusions were the same to the ones using the Q10 threshold (see Appendix).

To evaluate results stability, we performed the assembly of the raw data twice, using the pipeline described above and the reference genome assembly and using a de novo assembly as performed in PyRAD (Eaton, 2014). The parameters used for producing the SNP dataset based on de novo assembly were as follows: no mismatches between the bar codes of the two reads (Illumina paired‐end sequencing), base calls with a phred quality score below 20 were converted to Ns (undetermined sites) and reads including more than 4 Ns were discarded, minimum genotype depth 5, clustering threshold 0.90 and the remaining parameters kept as default. Our preliminary analyses on this dataset resulted in the same conclusions with the reference genome dataset, indicating that our results are stable regardless of genome assembly methods (e.g., see Appendix).

The final global dataset included 57,931 SNPs present on 6,867 scaffolds of the 154,782 scaffolds (Chen et al., 2015). These were the longest of the reference genome scaffolds (total length of the represented scaffolds; >10^9 ^bp). Two mosquitoes were excluded due to the poor sequencing quality, so the final global dataset consisted of 86 individuals. The software PGDSpider v. 2.0.5.2 (Lischer & Excoffier, 2012) was used to convert between file formats for downstream analyses.

### 2.3 Levels of *Ae. albopictus* differentiation and evolutionary relationships

To quantify levels of genetic differentiation between all population pairs, we estimated Fst values using Arlequin v.3.5 (Excoffier & Lischer, 2010) with 1,000 permutations (0.05 significance level). We then used one‐way ANOVA to compare the mean Fst values between different groups of populations.

To ascertain how many groups of genetically distinct populations occurred, we used a maximum‐likelihood (ML) approach implemented in the program ADMIXTURE (Alexander, Novembre, & Lange, 2009) and two multivariate methods: discriminant analysis (DA) of principal components—DAPC (Jombart, Devillard, & Balloux, 2010) and principal component analysis—PCA (Frichot & Francois, 2015) using the R packages adegenet and LEA, respectively. DAPC transforms the raw data using PCA and then performs a DA on the retained principal components to provide an efficient description of the genetic clusters using a few synthetic variables (discriminant functions). These variables are linear combinations of the original variables (raw data) that maximize the between‐group variance and minimize the within‐group variance (Jombart, Devillard, & Balloux, 2010). For ADMIXTURE analysis, we used reduced SNP datasets, as we filtered each one of the initial datasets (global, invasive, native) based on LD estimates, as recommended by the authors. Thus, we used the *r* ^2^_max_/2 value as a threshold, where *r* ^2^_max_ is the maximum squared correlation coefficient value estimated by VCFtools. This value was estimated based on a population (San Benedetto del Tronto, Italy; Table 1) of 36 individuals. This dataset was produced as described above, but due to the fragmented nature of the genome assembly, we selected only SNPs occurring in the 1,003 longest contigs of the genome (~25% of the genome's length in bp) to avoid a bias in our estimations. Thus, the final dataset on which we estimated the *r* ^2^max/2 value consisted of ~24K biallelic SNPs. To choose the correct value for *K* (number of genetic clusters), the ADMIXTURE's cross‐validation procedure was used. A geographic map of population admixture proportions was constructed based on the mean *Q* values (genetic admixture proportions) for each population, using the mapplots package in R v.3.1.3 (R Core Team 2013).

The genetic differentiation was quantified at three different levels using three SNP datasets (1) global [20 populations; in total 57,931 SNPs], (2) invasive [14 populations; in total 64,691 SNPs, of which 51,440 SNPs were common with the global], and (3) native [five populations; in total 64,245 SNPs, of which 35,843 were common with the global].

To evaluate the evolutionary relationships among populations, we used a ML approach as implemented in RAxML (Stamatakis, 2014) using 1,000 bootstraps and the general time‐reversible (GTR) model of evolution along with the CAT approximation of rate heterogeneity. We performed two ML analyses (1) using the global dataset and (2) using only individuals from the native range. We used the string “ASC_” to apply an ascertainment bias correction to the likelihood calculations, and the standard correction by Paul Lewis (Lewis, 2001), when only variant sites are included in the data set, following the software manual. For this analysis, we identified candidate SNPs under selection and excluded them as neutrality is one assumption of these methods. We used two methods to detect outlier loci, one based on multivariate analysis and implemented in R using the pcadapt package (Luu, Bazin, & Blum, 2017) and another based on Fst values between populations and implemented in the program BayeScan (Foll & Gaggiotti, 2008). In both methods, we considered *Q* values lower than 0.05 for outlier's detection. The pcadapt does not require grouping individuals into populations (Luu, Bazin, & Blum, 2017). As BayeScan is a population‐based approach, we ran it on two data sets, on all 20 populations separately and on two groups according to their ability to undergo facultative diapause, a trait known to be under strong selection [for a review, see (Armbruster, 2016)]. Given that a different number of SNPs were detected by each program and that only a small number of SNPs were common, we conservatively excluded from the phylogenetic analyses all the candidate SNPs (in total 7,576 SNPs) detected by at least one method. We are aware that given the false‐positive rate associated with these types of analyses (Luu, Bazin, & Blum, 2017), we also may have excluded SNPs that were not under selection. However, this possibility is unlikely to bias our analyses, given the large number of SNPs in the final dataset (50,335 SNPs).

### 2.4 Levels of *Ae. albopictus* diversity

We estimated individual observed heterozygosity (Ho) using VCFtools and dividing the number of heterozygous loci by the number of genotyped loci in each individual. Based on Trucchi et al. (2016), a number of parameters were taken into account in the Ho estimations. First, to ensure that we do not include nonorthologous loci with artificially high heterozygosity, we used a reference genome alignment. Second, to deal with the relationship between depth coverage and the possibility of detecting heterozygosity, we further filtered our datasets to decrease the depth (DP) range between genotypes (10X < DP < 60X) and increase the minimum DP threshold as higher DP values lead to more accurate genotype calls. We then tested for a linear relationship between individual Ho and individual mean DP of the loci (*R* ^2 ^< 0.27 in all cases). Finally, we grouped the samples based on the sampling locality or their geographic group and compared the mean Ho per group among the following groups: (1) global [86 individuals; 15,402 SNPs], (2) native [23 individuals; 19,468 SNPs], (3) invasive [63 individuals; 17,497 SNPs], (4) Cluster1 (see Results) identified by ADMIXTURE [47 individuals; 21,806 SNPs], and (5) Cluster2 (see Results) identified by ADMIXTURE [39 individuals; 18,613 SNPs]. In each dataset, only loci present in at least 70% of the individuals were included.

The nonparametric Kruskal–Wallis test was used to compare the mean heterozygosity between the populations as the small sample size per population (three to six individuals) did not meet the assumptions of the parametric tests. One‐way ANOVA was used to compare the means between specified group regions. In all cases, only populations that did not differ in their mean heterozygosity (*p* > .05) were grouped together in the same region. When the ANOVAs identified statistically significant differences, we implemented a post hoc Tukey test to detect the specific groups that differ in their mean heterozygosity.

## 3 RESULTS

### 3.1 Marker discovery and descriptive statistics on the SNP datasets

After quality filtering, the sequencing of the ddRAD libraries resulted in ~13.0–32.0 million reads per mosquito. A total of 5,145,180 SNPs were obtained after mapping to the reference genome and filtering for Q10 mapping quality. Further filtering (5.0X minimum genotype depth; presence in at least 70% of the individuals; MAF > 0.05) resulted in 57,931 biallelic SNPs for the global dataset. These SNPs were in the 6,867 scaffolds, which encompass ~58% of the total reference genome size (bp). The average sequence depth per individual was 16.04X ± 6.42X (SD), and the average coverage per site was 16.04X ± 6.64X (SD). The amount of missing data on a per individual was 16.15% ± 11.8% (SD) and on a per‐site basis 16.15% ± 8.44% (SD). The invasive dataset (63 individuals; 64,691 biallelic SNPs) had an average sequence depth per individual of 16.30X ± 6.75X (SD) and an average coverage per site of 16.30X ± 6.83X (SD). The native dataset (23 individuals; 64,245 biallelic SNPs) had an average sequence depth per individual of 15.04X ± 5.42X (SD) and an average coverage per site of 15.05X ± 6.18X (SD). The amount of missing data on a per‐individual basis (invasive; 16.00% ± 12.00% and native; 15.18% ± 11.4%) as well as on a per‐site basis (invasive; 16.00% ± 8.44% and native; 15.18% ± 8.23%) was similar to the global dataset.

### 3.2 Levels of genomic differentiation

Table 2 summarizes estimates of genomewide differentiation for all populations and Figure 2a summarizes the average Fst values within and between continents. Fst values between sites within its native Asian range are on average lower (one‐way ANOVA; *p* = .002) than the Fst values between sites from the same or different continents from the invasive range, although in both ranges, two genetic groups are present (Cluster1 and Cluster2), as indicated by the Admixture analysis in the global dataset (see Results below).

**Table 2:** Fst values for all pairs of *Aedes albopictus* populations as estimated by Arlequin (Excoffier & Lischer, 2010) for the 69,046 SNP dataset retrieved from the reference genome assembly

	1	2	3	4	5	6	7	8	9	10	11	12	13	14	15	16	17	18	19
1. VIET																			
2. BRO	0.232																		
3. COAT	0.144	0.252																	
4. CORP	0.089	0.145	0.118																
5. DRC	0.137	0.277	0.204	0.146															
6. FCV	0.080	0.237	0.155	0.092	0.115														
7. GRE	0.085	0.219	0.152	0.089	0.136	0.079													
8. HAW	0.092	0.196	0.152	**0.032**	0.155	0.102	0.097												
9. ITA	0.087	0.193	0.131	0.034	0.141	0.093	0.078	0.051											
10.ILAB	0.228	0.311	0.262	0.169	0.278	0.236	0.205	0.184	0.131										
11.BER	0.127	0.190	0.161	0.048	0.187	0.138	0.122	0.091	0.077	0.202									
12.KAG	0.111	0.198	0.140	0.046	0.165	0.116	0.109	0.074	0.043	0.191	0.084								
13.KLP	0.068	0.232	0.144	0.087	0.118	0.060	0.079	0.098	0.088	0.236	0.131	0.111							
14.VIRG	0.094	0.164	0.114	**0.011**	0.151	0.106	0.092	0.056	0.030	0.167	0.045	0.030	0.101						
15.NEW	0.110	0.173	0.127	0.025	0.164	0.120	0.108	0.077	0.048	0.182	0.073	0.044	0.116	**0.001**					
16.FLO	0.121	0.184	0.151	0.039	0.172	0.131	0.121	0.086	0.082	0.213	0.082	0.089	0.116	0.060	0.082				
17.PRES	0.211	0.319	0.110	0.176	0.272	0.225	0.226	0.212	0.199	0.319	0.231	0.217	0.217	0.191	0.195	0.220			
18.SALV	0.170	0.292	0.113	0.151	0.231	0.183	0.183	0.181	0.167	0.296	0.202	0.177	0.167	0.158	0.171	0.163	0.172		
19.SEN	0.057	0.204	0.131	0.062	0.108	0.057	0.048	0.075	0.053	0.197	0.106	0.079	0.045	0.066	0.089	0.102	0.196	0.153	
20.TOK	0.104	0.187	0.132	0.035	0.159	0.113	0.106	0.075	0.042	0.175	0.079	0.035	0.102	0.018	0.034	0.085	0.195	0.146	0.083

Non significant values (*p *>* *.05) are indicated by bold characters. Population codes as in Table 1.

### 3.3 Pattern of genomic differentiation

**Figure 3:**
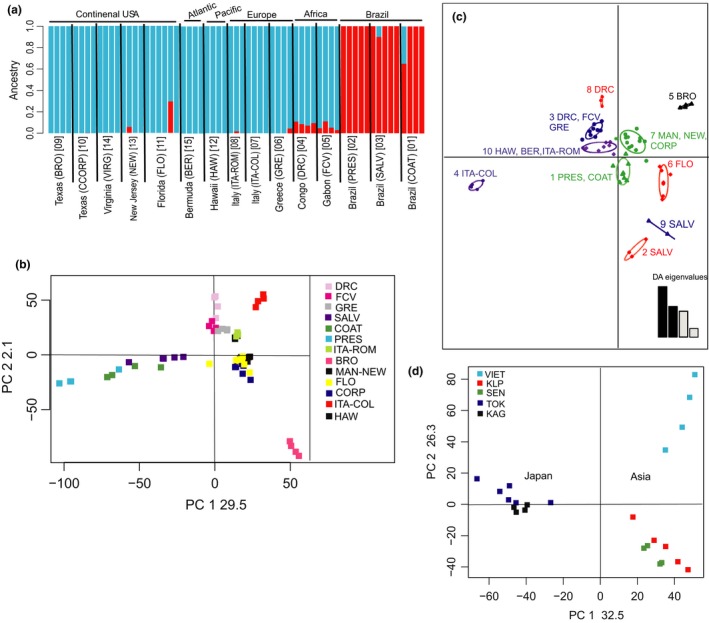
(a) ADMIXTURE barplot for *K* = 2 (best supported) for the *Aedes albopictus* populations from the invasive range using the invasive dataset (64,691 SNPs). For details, see legend in Figure 2B. Principal components analysis (PCA) on the invasive (b) and the native (d) range of populations as implemented and plotted in LEA package, presenting the projection of all individual mosquitoes on the first two PCs and obtained using the respective dataset. (c) Discriminant analysis of principal components (DAPC) for the *Aedes albopictus* populations from the invasive range considering ten DAPC groups obtained using the invasive dataset. The graph represents the individuals as dots and the groups as inertia ellipses. A barplot of eigenvalues for the discriminant analysis (DA eigenvalues) is displayed in the inset. The number of bars represents the number of discriminant functions that retained in the analysis, and the eigenvalues correspond to the ratio of the variance between groups over the variance within groups for each discriminant function

Figure 2b shows the results of the Admixture analysis using 37,707 biallelic unlinked SNPs for the global data set. The Admixture analysis only on the invasive dataset is presented in Figure 3a. The Admixture analysis on the native dataset is not presented as *K* = 1 was supported as the best run.

Admixture analysis for the global dataset supported the existence of two genetically distinct clusters, each including populations from the native and the invasive range. Cluster1 (red, Figure 2b) consists of populations from Japan (native range) together with Italy, Bermuda, and USA (invasive range). Cluster2 (blue, Figure 2b) includes samples from Singapore, Malaysia, and Vietnam (native range) together with Africa, Greece, and Brazil (invasive range). Within each cluster, Fst values among populations are slightly higher in Cluster2 than in Cluster1 (ANOVA; *p* = .006). The next best clustering for *K* = 3 is presented as pie charts in Figure 2c. Most populations within each cluster include individuals with high assignment to the respective cluster (*Q* values > 0.75). However, some samples from both the native (Singapore and Vietnam) and invasive (Greece, Italy, Hawaii, and Florida) ranges show evidence of genetic admixture. Several individuals have low mean *Q* values (for *K* = 2; Greece 0.61–0.70, Hawaii 0.68–0.90, Singapore 0.67–0.69, Vietnam 0.77–0.82 and for *K* = 3; Italy 0.71–0.76, Hawaii 0.51–0.84), suggesting that they could be the results of recent matings between individuals belonging to the two different clusters or retention of shared ancestral polymorphisms.

**Figure 4:**
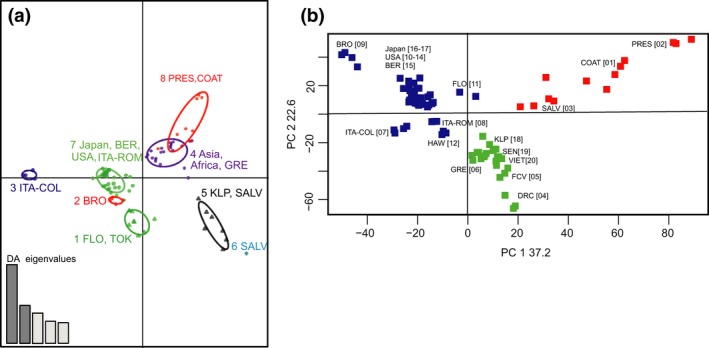
(a) Discriminant analysis of principal components (DAPC) for all *Aedes albopictus* populations considering eight DAPC groups. The graph represents the individuals as dots and the groups as inertia ellipses. A barplot of eigenvalues for the discriminant analysis (DA eigenvalues) is displayed in the inset. For details, see legend of Figure 3. (b) Principal components analysis (PCA) as implemented and plotted in LEA package, presenting the projection of all individual mosquitoes on the first two PCs. The colors of the three groups are consistent with those in Figure 2c for the three groups indicated by Admixture

Figure 4 shows the results of the DAPC and the PCA analysis on the global data set. The first DA axis in DAPC separated the same groups of populations included in Cluster1 and Cluster2, as defined by the Admixture analysis (Figure 2b). The DAPC also highlights the genetic distinction of one of the two Italian samples (San Benedetto del Tronto, ITA‐COL) and one of two samples from Texas, USA (Brownsville, BRO). The PCA (the first two axes explain 59.8% of the variance) broadly confirms the results from the other two analyses and highlights the genetic differentiation among Brazilian sampling sites (Figure 4b).

The genetic differentiation was also evident when invasive and native (Figure 3) populations were analyzed separately. For example, in the invasive range the DAPC identified additional partitioning among the populations (10 groups Figure 3c). This analysis also highlights the high level of differentiation between the Brazilian populations and the distinctiveness of Florida (FLO) and Texas (BRO) ones, compared to the other US populations (Figure 3c). For the native populations, the multivariate methods (Figure 3d) grouped the populations in the same two genetic clusters recovered when all samples were analyzed together, although ADMIXTURE supported *K* = 1 as the best run.

### 3.4 Evolutionary relationships

**Figure 5:**
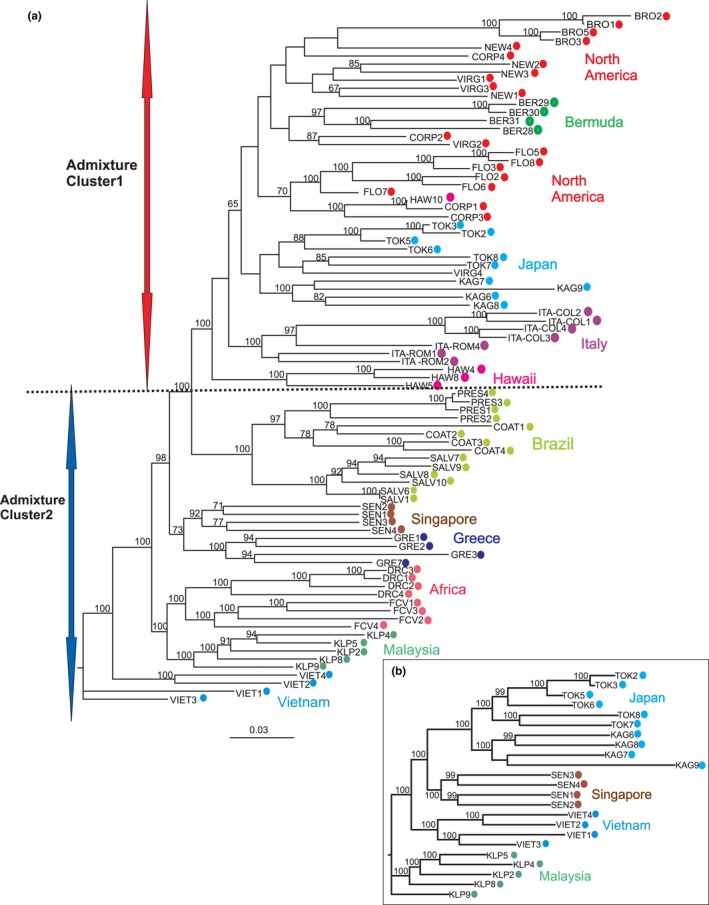
Phylogenetic relationships. Maximum‐likelihood unrooted phylogenetic trees reconstructed using ~50,000 SNP dataset. Tip labels are as in Table 1. Bootstraps percentages >65 are indicated on the nodes

We carried out ML phylogenetic analyses on all samples and only on the native ones (Figure 5). The unrooted ML phylogenetic tree on all samples confirmed the grouping of all the Cluster1 populations into one highly supported clade (BS = 100), whereas the remaining populations (Cluster2) formed highly supported clades based on their origin (e.g., Brazil, Africa, and Malaysia; Figure 2). Figure 5b shows the same ML analyses using only the native range samples, illustrating strong support for clades that include samples from the five geographic localities.

### 3.5 Levels of genomic diversity

**Figure 6:**
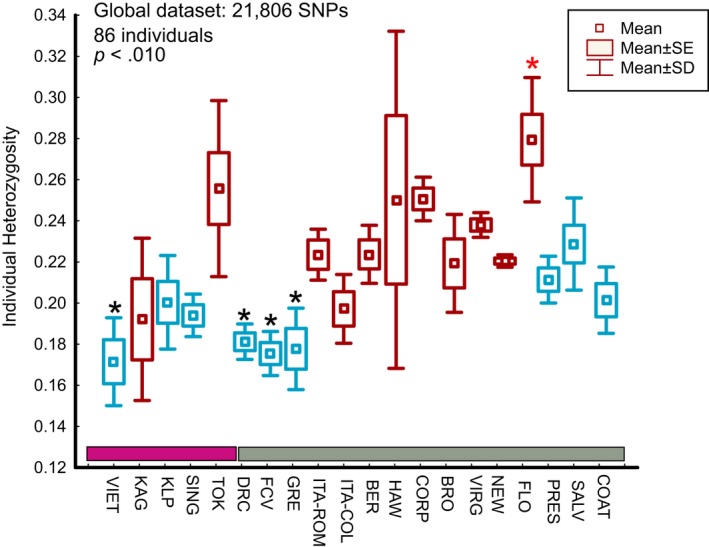
Genomic diversity. Individual observed heterozygosity per population as estimated using VCFtools for the global SNP datasets. Individuals then grouped by population. The mean, standard deviation (*SD*), and the standard error (*SE*) are presented. The nonparametric Kruskal–Wallis test was implemented to test for differences between populations (*p* < .05). The groups that differ significantly in their mean Ho are marked with differentially colored asterisk. Colors are consistent with the ones in Figure 2; thus, the bars above the *X* axis of the plot indicate the native (purple) and the invasive (gray) species range while the red‐ and the blue‐colored graphs represent the Clusters 1 and 2, respectively, as indicated with ADMIXTURE and plotted in Figure 2B. Population codes as in Table 1

Genetic diversity (Figure 6) was studied at five levels (global, invasive, native, Cluster1, and Cluster2). The mean observed heterozygosity (Ho) is slightly lower (Ho ~0.18) in Africa and Greece (invasive) compared with the native range populations (Ho ~0.21) but not statistically significant different. In general, we found no statistically significant differences in the mean Ho levels between the invasive and native populations with the exception of samples from Florida, Hawaii, and Brazil, which have statistically higher heterozygosity levels than the ones from Africa, Greece, and south Asia. The same overall results were obtained when samples were grouped according to geographic regions and the mean Ho was compared through one‐way ANOVA (ANOVA; *p* < .04 for global dataset; Hawaii–USA higher than Africa–Greece–Asia, invasive dataset; Hawaii–USA–Brazil higher than Africa–Greece–Asia, Cluster2 dataset; Brazil higher than Africa–Greece–Asia).

## 4 DISCUSSION

Over the last 30 years, *Ae. albopictus* has spread from it native Asian range to all continents except Antarctica, making it one of the most invasive mosquitoes on the planet (Benedict, Levine, Hawley, & Lounibos, 2007; Lounibos, 2002). Here, we describe the development of genomewide SNP markers and demonstrate for the first time that these high‐resolution markers are able to detect fine‐scale genetic structure across the worldwide distribution of *Ae*. *albopictus*.

### 4.1 Genomewide SNP marker discovery

The implementation of the ddRAD sequencing enabled us to identify ~58,000 biallelic SNPs across the genome of *Ae*. *albopictus* after the filtering process (>210,000 SNPs applying only genotype DP > 5.0X and presence in at least 70% of the samples filter). As the ddRAD tags are randomly distributed across the genome, this method makes it possible to affordably screen a large number of genomic regions in many samples. Despite the fact that only a small percentage (~6%) of the reference genome scaffolds are represented in our datasets, these are the longest scaffolds, covering more than 1 × 10^9 ^bp (out of the ~1.9 × 10^9 ^bp). This suggests that the small proportion of scaffolds in our dataset is due to the fragmented nature of the reference genome (~154,000 scaffold count) rather than to a bias with our dataset.

The use of two REs during the ddRAD library preparation provided consistency in markers recovery (~58,000 SNPs with coverage in at least 70% of the individuals on a global scale and on average ~16% missing data). This consistency in marker recovery increases the possibility of retrieving the same loci to be sequenced across all the individuals and reduces the amount of missing data compared with other similar methodologies that use only one restriction enzyme [i.e., RAD‐tags (Baird et al., 2008)]. Moreover, these SNP markers can be used as baseline for future studies that include additional samples worldwide or focus on samples from a specific geographic region, as the same SNPs can be used and data can be combined much more easily than for studies based on other markers like microsatellite loci or mtDNA [for a review see (Goubert, Minard, Vieira, & Boulesteix, 2016)].

### 4.2 Global genetic differentiation

The 20 native and invasive *Ae. albopictus* population samples group into at least two distinct genetic clusters that are broadly consistent with inferences based on ecophysiological traits, such as photoperiodic diapause and the cold tolerance of eggs (Cluster1 and Cluster2; Figures 2, 3, 4, and 5). The genetic connection between USA, Italy, and Japan (Cluster1) has also been shown by allozymes (Urbanelli, Bellini, Carrieri, Sallicandro, & Celli, 2000) and mtDNA (Birungi & Munstermann, 2002) data. This genetic similarity corroborates the hypothesis that Japan was the origin of colonists in North America (Birungi & Munstermann, 2002; Dalla Pozza, Romi, & Severini, 1994; Kennedy, 2002) and that North American populations were the source of the first invasive populations detected in Italy (Dalla Pozza et al., 1994; Urbanelli et al., 2000). No previous information exists regarding the source of invasion into Bermuda. Our data imply a North American origin likely due to the geographic proximity of Bermuda to North America and/or the frequent travel and commerce between these two areas. Although the Hawaiian samples grouped within Cluster1 (Figure 2), they are genetically distinct (Figures 3d and 4b) from the Japanese and the North American samples (Fst = 0.03–0.19; Table 2). Their evolutionary distinctiveness is also supported by their clade placement in the ML tree (Figure 5). This, together with the fact that the Hawaiian samples also show clear signs of genetic admixture while the continental US ones do not (Figure 2), suggest that the *Ae. albopictus* colonization of Hawaii and the continental USA are likely to have occurred independently and that Hawaii was colonized multiple times from different regions.

For Cluster2, the genetic clustering of populations from Southeast Asia and South America is consistent with the observation that populations from both regions lack a photoperiodic diapause response (Lounibos, Escher, & Lourenço‐De‐Oliveira, 2003). However, the biogeographic relationship of the South American populations relative to the remaining *Ae. albopictus* populations cannot be unequivocally inferred (Figure 5), because the Admixture analyses placed the Brazilian populations within Cluster2, while the phylogenetic analysis clusters them in a single well supported clade sister to the clade including all the populations from Cluster1. However, regardless of their origin, the lack of evidence for genetic admixture (average *Q* values > 0.90) and the fact that they appear as a monophyletic group (Figure 5) suggest that the Brazilian samples were derived from a single *Ae. albopictus* invasion from a native population, presumably a nondiapausing population in Southeast Asia, that was not sampled in this study. The African‐Continental Asia grouping (Figure 2) was also suggested in a previous mtDNA analysis (Kamgang et al., 2013). The clustering of the Greek samples with samples from Africa and Asia (Figures 2, 3, 4 and 5) contradicts a mtDNA analysis, which supported the clustering of Greek samples with the Hawaii–USA group (Kamgang et al., 2013). However, as our samples from these regions were collected a few years later than the ones used in the Kamgang et al. (2013) study, we cannot rule out the possibility that the discrepancy between the two studies could be due to the temporal structure of the populations. Our results are consistent with microsatellite data suggesting that *Ae*. *albopictus* from Greece and Italy are genetically different (Manni et al., 2017). This implies that Europe was invaded at least two times from genetically distinct *Ae. albopictus* populations. Additionally, the populations from Greece, Brazil, and Singapore share the same F1534C kdr mutation. This mutation may confer resistance to pyrethroids and DDT insecticides (Aguirre‐Obando, Martins, & Navarro‐Silva, 2017; Kasai et al., 2011; Xu et al., 2016). Other substitutions (F1534L and F1534S) in the same kdr codon were found in the USA (Marcombe, Farajollahi, Healy, Clark, & Fonseca, 2014; Xu et al., 2016) and China (Chen et al., 2016; Xu et al., 2016). The geographic distributions of these mutations raise questions of whether they emerged in native regions and colonized new areas or arose independently de novo in several places. Our grouping of populations (Brazil–Greece–Singapore vs USA) is consistent with the finding of different kdr mutations in the two groups, highlighting the importance of studying the genetic structure in designing vector‐control strategies as knowing the source of a given invasion contributes in predicting possible resistance to insecticides.

### 4.3 Genetic differentiation within the native region

Our analyses included five Asian samples that cluster into two genetically distinct groups (Figures 2 and 3). Interestingly, populations from Singapore and Vietnam show signs of genetic admixture, which is in agreement with mtDNA studies (Maynard et al., 2017; Zhong et al., 2013) and suggests ongoing genetic exchange likely with mosquitoes from localities not sampled in this study. A previous study has established that Cluster1 and Cluster2 populations are fully interfertile (O'Donnell & Armbruster, 2009).

The phylogenetic analyses show that all individuals from the same geographic location for these five native sampling sites cluster in monophyletic groups (Figure 5B), reinforcing the results of the Admixture and multivariate analyses. The phylogenetic analyses also showed that the Southeast Asian native populations do not group together in a single clade but each one groups with a different invasive population (e.g., Malaysia–Africa, Japan–USA, Singapore–Greece; Figure 5).

Interestingly, a recent microsatellite survey using 17 microsatellite loci and 10 worldwide populations, including three native samples (China, Thailand, and Japan), did not find the genetic differentiation within the native Asian range (Manni et al., 2017) that we show in this study. This limited their capacity to assign the origin of the invasive samples to specific geographic regions and led them to conclude that the human‐aided dispersal of *Ae*. *albopictus* out of Asia was “chaotic.” Our analyses on the other hand reveal a strongly supported genetic structure both within the native and the invasive range, and at the same time, the strong connection between distant geographic regions (e.g., Malaysia–Africa, Japan–USA) reveals the human‐mediated transport. The discrepancy between this study and that of Manni et al. (2017) could be due to differences in the relative power of the two types of markers used (microstatellite loci vs tens of thousands of SNPs), given that the genetic differentiation between Malaysia–Singapore and USA–Hawaii was also not detected in another microsatellite analysis (Maynard et al., 2017). Of particular note is the difference in genetic differentiation of the Japanese populations in the two studies. In the microsatellite study (Manni et al., 2017), the Japanese sample (Nagasaki, on the southern island of Kyushu) is genetically admixed and not distinguishable from the other two Asian continental samples used in that study. On the other hand, in our analyses the two Japanese samples are closely related to each other and genetically distinct from the Asian continental samples (Figures 2 and 3). These results emphasize the advantages of using a large number of genetic markers distributed across the genome for identifying fine‐scale differentiation. When combined with thorough population sampling, this high level of resolution is particularly valuable in the context of studying the phylogeography of an invasive and medically important disease vector because it has important implications for inferring routes of invasion, the potential for insecticide resistance, and vector competence in invasive populations.

### 4.4 Genetic differentiation within the invasive regions

The high‐resolution power of the SNPs described here is also illustrated by the genetic differentiation detected among populations from within invasive regions. For example, the multivariate analysis shows that the Brownsville, TX, and to a smaller extent Florida samples are distinct from other eastern North American populations. The multivariate analysis also shows a clear genetic distinction (Figure 4) between the South American populations (SALV from PRES‐COAT) on a north to south basis. Similarly, the two Italian populations from western (ROM, Rome) and eastern (COL, San Benedetto del Tronto) central Italy (Figure 2c) are quite differentiated (Fst = 0.13). Finally, the two continental African populations from Gabon and Congo are genetically distinct (Fst = 0.12, Figure 2). In all of these cases, the populations from within the same region (USA, South America, Italy, Africa) are in the same cluster (Figure 2b) and clade (Figure 5), suggesting divergence of populations within regions due to in situ differentiation following either a single invasion event or multiple invasions from genetically distinct populations but from the same broad geographic area (Figures 2 and 3). More intensive population sampling will be required to resolve these possibilities.

### 4.5 Genetic diversity

The observed heterozygosity (Ho) varies among population and sampling regions (Figure 6). Within the native range, populations do not differ in genomic diversity with possibly the exception of the samples from Tokyo and Vietnam (Tokyo had marginally significant higher Ho than Vietnam in the native dataset analysis). In contrast, within the invasive range, the populations from Greece, Africa, and the Italian laboratory colony have lower Ho levels than the population from Florida. Although the lower Ho in the colony is expected, the difference in levels of diversity between the samples from Greece and Africa (Cluster2) and the US populations may be a consequence of differences in their invasion history. For instance, the high Ho levels found in the Hawaiian samples could be due to the relatively old age of the island colonization [>100 years ago, (Lounibos, 2002)] and the possibility of multiple invasions, given the levels of admixed ancestry found in this population (Figure 2).

In general, we do not see significant differences in levels of genomic diversity between native versus invasive samples. This could be due to the lack of sampling depth in the study or reflect the colonization of new areas by a large number of propagules that retained the levels of diversity of the source population(s), as has been proposed to explain the lack of a genetic signature of “founder effects” for the US *Ae. albopictus* invasive populations (Kambhampati, Black, Rai, & Sprenger, 1990). Future analyses with wider spatial coverage would be necessary to have the statistical power to distinguish between alternative invasion scenarios (single vs multiple invasions; large vs small propagules; old vs recent invasions; ongoing vs past gene flow), which most likely would be specific to each invasion.

## 5 CONCLUSIONS

We identified tens of thousands of SNPs common to globally distributed populations of *Ae. albopictus* and used them to provide baseline data on the evolutionary history of this highly invasive vector species. The evolutionary scenario emerging from our analyses confirms the results of other studies in describing a complex invasion history with multiple independent invasions and/or invasion of a large number of colonists from both native and previously invaded areas, followed in some cases by genetic intermixing between samples from different genetic backgrounds. Our results are novel because the power of ~58,000 SNPs distributed across the genome enabled detection of differentiation within regions that was not revealed in studies using less powerful genetic markers. These findings have profound implications for control and monitoring of this important disease vector, as they can reveal the mosquitoes' mode of transportation, the likelihood of recurrent invasions and help predict the chance of establishment based on climate matching between source and invasive localities. Furthermore, different regions of the world use different insecticides, so identifying invasion sources can help predict the effectiveness of insecticides due to resistance in the source population(s). Additionally, as *Ae. albopictus* populations vary in viral competence (Lourenco de Oliveira, Vazeille, de Filippis, & Failloux, 2003), so understanding whether an introduction is from an active transmission region helps assess the public health threat of the invasion.

Last, the wealth of genomewide markers across a global spatial scale provided by this work could be used as a baseline for future association studies aimed at understanding the genetic basis of complex traits, including key ecological adaptations (e.g., diapause) and traits related to disease transmission, which can then be used to develop new strategies and targets for vector control. The availability of a common set of tens of thousands of variable genome markers enables independent studies to be combined and thus to take advantage of cumulative knowledge and data.

## DATA ACCESSIBILITY

The datasets used and/or analyzed during the current study are available from the PI of the study (AC) on reasonable request.

## CONFLICT OF INTEREST

None declared.

## AUTHORS' CONTRIBUTIONS

PK carried out part of the molecular laboratory work, data analysis, and sequence alignments and drafted the first version of the manuscript. JBR carried out part of the molecular laboratory work and part of the data analysis and helped draft the manuscript; VP carried out part of the molecular laboratory work and helped draft the manuscript; PAA GF SU AJM provided samples and revised the manuscript critically for important intellectual content; AC and PAA conceived of the study, designed and coordinated the study, and helped draft the manuscript. All authors gave final approval for publication.
