# Supplementary material for: Population genomics of the Asian tiger mosquito, Aedes albopictus: insights into the recent worldwide invasion
Source: Ecol Evol. 2017 Oct 24;7(23):10143–57. doi: 10.1002/ece3.3514 (PMC5723592; doi:10.1002/ece3.3514)
Supplement: Supplementary file 1 [file ECE3-7-10143-s001.docx]

**Supplementary File**

**Preliminary analyses**

***A. De novo* assembly using pyrad**

The raw sequences were retrieved as mentioned in the Material and Methods section, aligned using the *de novo* assembly (DN) pipeline as implemented in pyRAD v.3.04 with the “pairddrad” option for pair-end sequencing of ddRAD data (Eaton 2014). Base calls with a phred quality score below 20 were converted to Ns (undetermined sites) and reads including more than four Ns were discarded. However in the *de novo* assembly, the “clustering threshold” and the “minimum depth of coverage” are crucial parameters that can cause either under- or over-merging of clusters. So following a similar reasoning used by Razkin et al. (2016) and Viricel et al. (2014), we tested different sets of parameters (clustering threshold; 0.85, 0.90 and 0.94; minimum depth; 5 and 10; minimum coverage; 75%, 80%, 90% of the samples) and we finally set the clustering threshold to 0.90, the minimum depth to five, and the minimum coverage to 90% of the samples. The raw sequence reads were de-multiplexed and quality filtered using the same program. This dataset consisted of 76,269 SNPs.

We studied the genetic structure of the populations using the same software as used in the reference genome assembly. To comply with the requirement of ADMIXTURE to use unlinked loci, we used the unlinked SNPs dataset provided by pyrad. This dataset is a random sample of one SNP per locus and consisted of 3,955 SNPs. The result of the ADMIXTURE analysis is presented in **Figure S1** while the pair-wise Fst values for all the 20 populations used in this study as estimated in Arlequin is presented in **Table S1**. For convenience in comparison, we present the Fst values from both the reference genome assembly and the *de novo* assembly combined in one table.


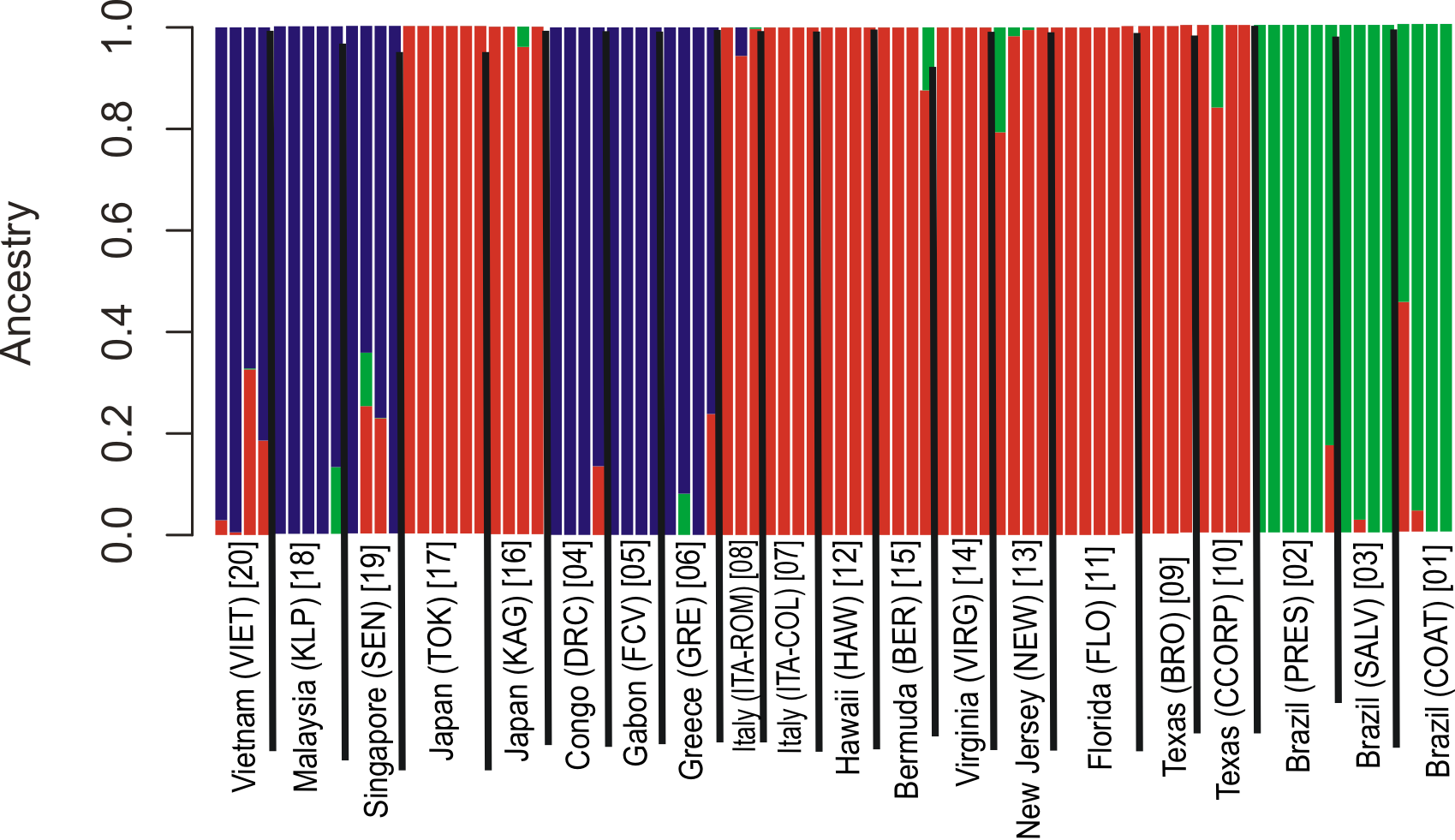


**Figure S1.** Genetic structure analysis as performed in ADMIXTURE for the *de novo* dataset produced by pyrad for K=3. Individuals are vertical bars along the plot. The Y axis represents the percentage of each individual (Q value) assigned to a cluster, the height of each color represents the probability of assignment to a genetic cluster. The black vertical lines indicate population limits.

**Table S1**: Fst values for all pairs of *Ae. albopictus* populations as estimated by Arlequin (Excoffier & Lischer 2010) for the 76,269 SNPs dataset retrieved from the *de novo* assembly using pyrad (above diagonal) and those of the reference genome assembly as presented in Table 2 (below diagonal). Non significant values (p>0.05) are indicated by bold characters. Population codes as in Table 1.

|  | **1** | **2** | **3** | **4** | **5** | **6** | **7** | **8** | **9** | **10** | **11** | **12** | **13** | **14** | **15** | **16** | **17** | **18** | **19** | **20** |
| --- | --- | --- | --- | --- | --- | --- | --- | --- | --- | --- | --- | --- | --- | --- | --- | --- | --- | --- | --- | --- |
| **1.VIET** |  | 0.214 | 0.143 | 0.088 | 0.133 | 0.091 | 0.089 | 0.105 | 0.077 | 0.218 | 0.117 | 0.095 | 0.072 | 0.089 | 0.111 | 0.139 | 0.196 | 0.162 | 0.069 | 0.115 |
| **2. BRO** | 0.232 |  | 0.241 | 0.168 | 0.278 | 0.209 | 0.210 | 0.235 | 0.205 | 0.327 | 0.209 | 0.198 | 0.198 | 0.161 | 0.179 | 0.216 | 0.324 | 0.301 | 0.175 | 0.205 |
| **3. COAT** | 0.144 | 0.252 |  | 0.136 | 0.197 | 0.141 | 0.140 | 0.178 | 0.149 | 0.271 | 0.174 | 0.135 | 0.138 | 0.123 | 0.145 | 0.189 | 0.103 | 0.111 | 0.124 | 0.156 |
| **4. CORP** | 0.089 | 0.145 | 0.118 |  | 0.153 | 0.091 | 0.089 | 0.082 | 0.057 | 0.199 | 0.064 | 0.056 | 0.090 | **0.023** | 0.044 | 0.073 | 0.198 | 0.189 | 0.068 | 0.041 |
| **5. DRC** | 0.137 | 0.277 | 0.204 | 0.146 |  | 0.102 | 0.140 | 0.172 | 0.140 | 0.277 | 0.182 | 0.161 | 0.113 | 0.151 | 0.180 | 0.188 | 0.263 | 0.233 | 0.117 | 0.170 |
| **6. FCV** | 0.080 | 0.237 | 0.155 | 0.092 | 0.115 |  | 0.085 | 0.113 | 0.096 | 0.219 | 0.126 | 0.100 | 0.068 | 0.109 | 0.117 | 0.144 | 0.200 | 0.174 | 0.060 | 0.116 |
| **7. GRE** | 0.085 | 0.219 | 0.152 | 0.089 | 0.136 | 0.079 |  | 0.130 | 0.082 | 0.213 | 0.126 | 0.100 | 0.077 | 0.099 | 0.111 | 0.148 | 0.206 | 0.174 | 0.054 | 0.114 |
| **8. HAW** | 0.092 | 0.196 | 0.152 | **0.032** | 0.155 | 0.102 | 0.097 |  | 0.089 | 0.220 | 0.129 | 0.111 | 0.108 | 0.094 | 0.126 | 0.139 | 0.236 | 0.213 | 0.101 | 0.108 |
| **9. ITA** | 0.087 | 0.193 | 0.131 | 0.034 | 0.141 | 0.093 | 0.078 | 0.051 |  | 0.154 | 0.098 | **0.085** | 0.090 | 0.057 | 0.083 | 0.126 | 0.207 | 0.187 | 0.069 | 0.080 |
| **10.ILAB** | 0.228 | 0.311 | 0.262 | 0.169 | 0.278 | 0.236 | 0.205 | 0.184 | 0.131 |  | 0.210 | 0.213 | 0.200 | 0.169 | 0.197 | 0.249 | 0.335 | 0.321 | 0.190 | 0.202 |
| **11.BER** | 0.127 | 0.190 | 0.161 | 0.048 | 0.187 | 0.138 | 0.122 | 0.091 | 0.077 | 0.202 |  | 0.098 | 0.114 | 0.046 | 0.074 | 0.112 | 0.234 | 0.216 | 0.087 | 0.095 |
| **12.KAG** | 0.111 | 0.198 | 0.140 | 0.046 | 0.165 | 0.116 | 0.109 | 0.074 | 0.043 | 0.191 | 0.084 |  | 0.086 | 0.041 | 0.057 | 0.135 | 0.214 | 0.205 | 0.077 | 0.062 |
| **13.KLP** | 0.068 | 0.232 | 0.144 | 0.087 | 0.118 | 0.060 | 0.079 | 0.098 | 0.088 | 0.236 | 0.131 | 0.111 |  | 0.091 | 0.107 | 0.130 | 0.196 | 0.163 | 0.047 | 0.113 |
| **14.VIRG** | 0.094 | 0.164 | 0.114 | **0.011** | 0.151 | 0.106 | 0.092 | 0.056 | 0.030 | 0.167 | 0.045 | 0.030 | 0.101 |  | **0.011** | 0.086 | 0.194 | 0.181 | 0.076 | 0.039 |
| **15.NEW** | 0.110 | 0.173 | 0.127 | 0.025 | 0.164 | 0.120 | 0.108 | 0.077 | 0.048 | 0.182 | 0.073 | 0.044 | 0.116 | **0.001** |  | 0.123 | 0.195 | 0.213 | 0.100 | 0.058 |
| **16.FLO** | 0.121 | 0.184 | 0.151 | 0.039 | 0.172 | 0.131 | 0.121 | 0.086 | 0.082 | 0.213 | 0.082 | 0.089 | 0.116 | 0.060 | 0.082 |  | 0.253 | 0.230 | 0.123 | 0.115 |
| **17.PRES** | 0.211 | 0.319 | 0.110 | 0.176 | 0.272 | 0.225 | 0.226 | 0.212 | 0.199 | 0.319 | 0.231 | 0.217 | 0.217 | 0.191 | 0.195 | 0.220 |  | 0.181 | 0.182 | 0.217 |
| **18.SALV** | 0.170 | 0.292 | 0.113 | 0.151 | 0.231 | 0.183 | 0.183 | 0.181 | 0.167 | 0.296 | 0.202 | 0.177 | 0.167 | 0.158 | 0.171 | 0.163 | 0.172 |  | 0.151 | 0.192 |
| **19.SEN** | 0.057 | 0.204 | 0.131 | 0.062 | 0.108 | 0.057 | 0.048 | 0.075 | 0.053 | 0.197 | 0.106 | 0.079 | 0.045 | 0.066 | 0.089 | 0.102 | 0.196 | 0.153 |  | 0.099 |
| **20.TOK** | 0.104 | 0.187 | 0.132 | 0.035 | 0.159 | 0.113 | 0.106 | 0.075 | 0.042 | 0.175 | 0.079 | 0.035 | 0.102 | 0.018 | 0.034 | 0.085 | 0.195 | 0.146 | 0.083 |  |

**B. Effect of small sample size on the analyses**

To evaluate if the use of a sample size 4-6 is adequate in our study we performed a preliminary analysis on two populations, one from San-Benedetto Italy (16 individuals) and one from Greece (11 individuals). We performed the library preparation, the filtering and the snp-calling using the same pipeline as described in the Material and Methods section. For the filtering process we used vcftools and PLINK and these parameters: minimum genotype depth 7, only biallelic SNPs, maf 0.05, coverage 70% of samples, filtered for LD using PLINK and the r^2^max/2 value as a threshold. We retrieved a dataset of ~34,000 SNPs based on which we performed two basic population genetics analyses (a) estimation of individual heterozygosity and (b) identification of genetic structure using PCA and ADMIXTURE.

The results of these preliminary analyses are presented in **Figure S2** and indicate that decreasing the sample size from 11-16 to 4 does not change any of the conclusions we make about these two populations. More specifically, Admixture indicated K=2 as the best run in both cases, PCA indicated two groups using both datasets and the heterozygosity is different between the two populations (Kolmogorov-Smirnov test; p<0.05) in both datasets even though the confidence intervals in the pruned dataset are wider.


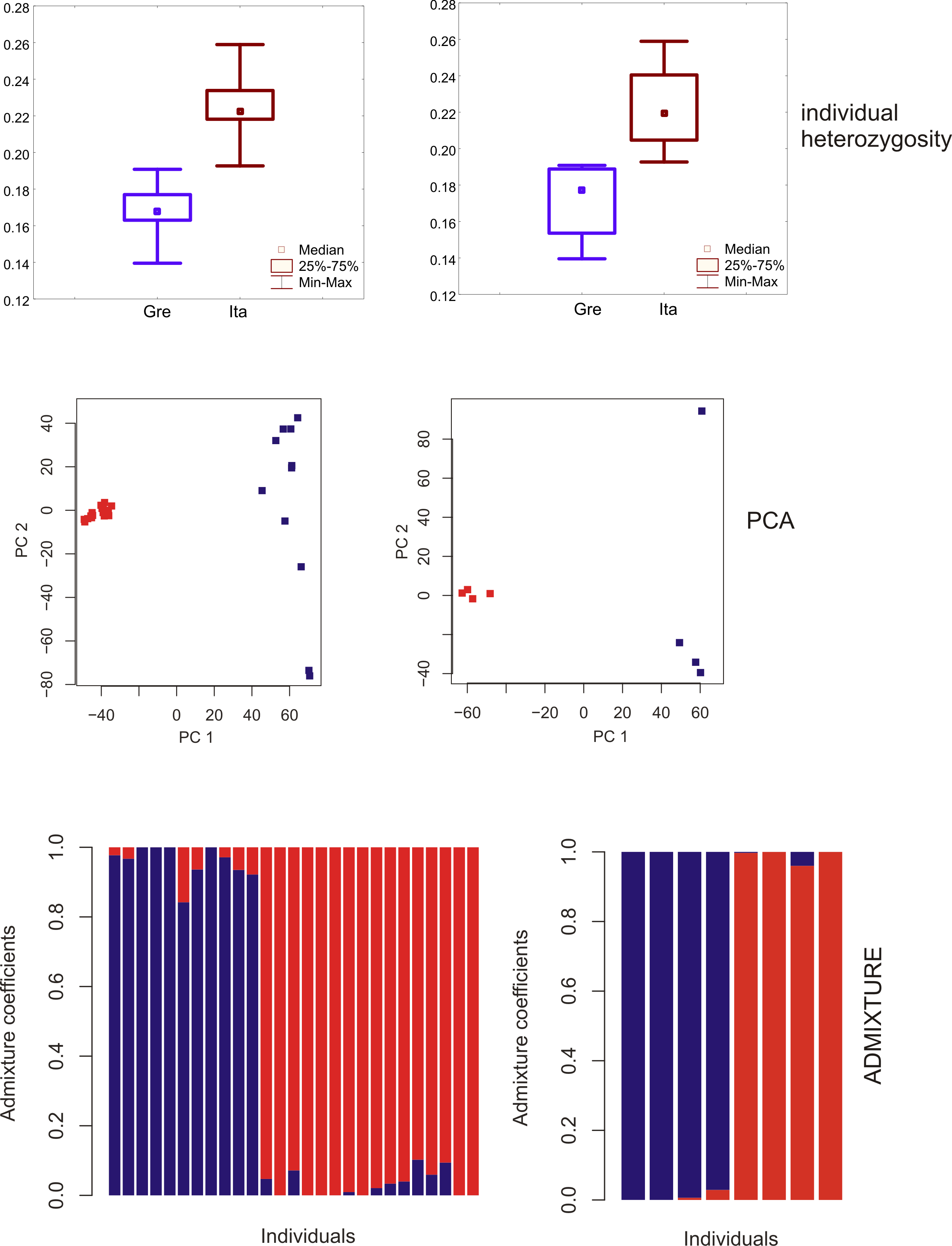


**Figure S2.** Real-case scenario testing the effect of sample size in our analyses. The analyses on the left column were performed using 16 samples from Italy (red in all graphs) and 11 samples from Greece (blue in all graphs) while the analyses on the right column were performed using 4 randomly selected samples from Italy (red in all graphs) and 4 from Greece (blue in all graphs).

**C. Effect of the mapping quality used in the study**

To evaluate the effect of using a minimum mapping quality of Q10 in order to exclude the inaccurate alignments from the downstream analyses, we used also a higher threshold (Q20) and we repeated the genetic structure analysis and the estimation of individual heterozygosity. The results are presented in **Figures S3** and **S4** indicating that this filtering had no severe effect on our conclusions but only on the number of SNPs retained.


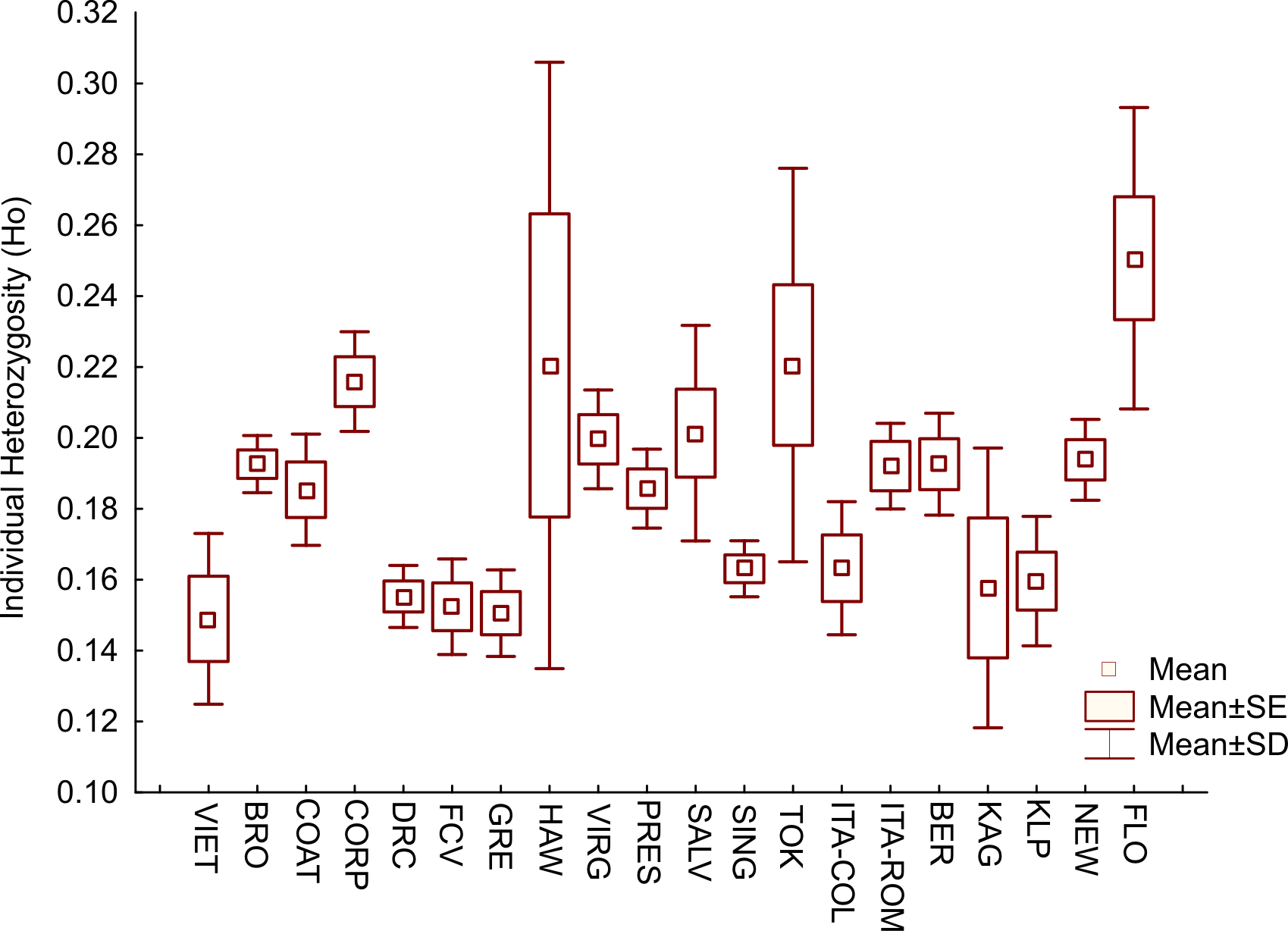


**Figure S3.** Individual heterozygosity (Ho) as estimated based on the reference genome assembly for the global dataset, retrieved with filtering the dataset for minimum mapping quality Q20. The remaining filtering parameters (minimum genotype depth, maf, coverage) were the same as they are presented in the Material and Methods section for the global dataset. Values of Ho were grouped together based on the population and the mean Ho compared between the populations using the Kruskal-Wallis non parametric test.


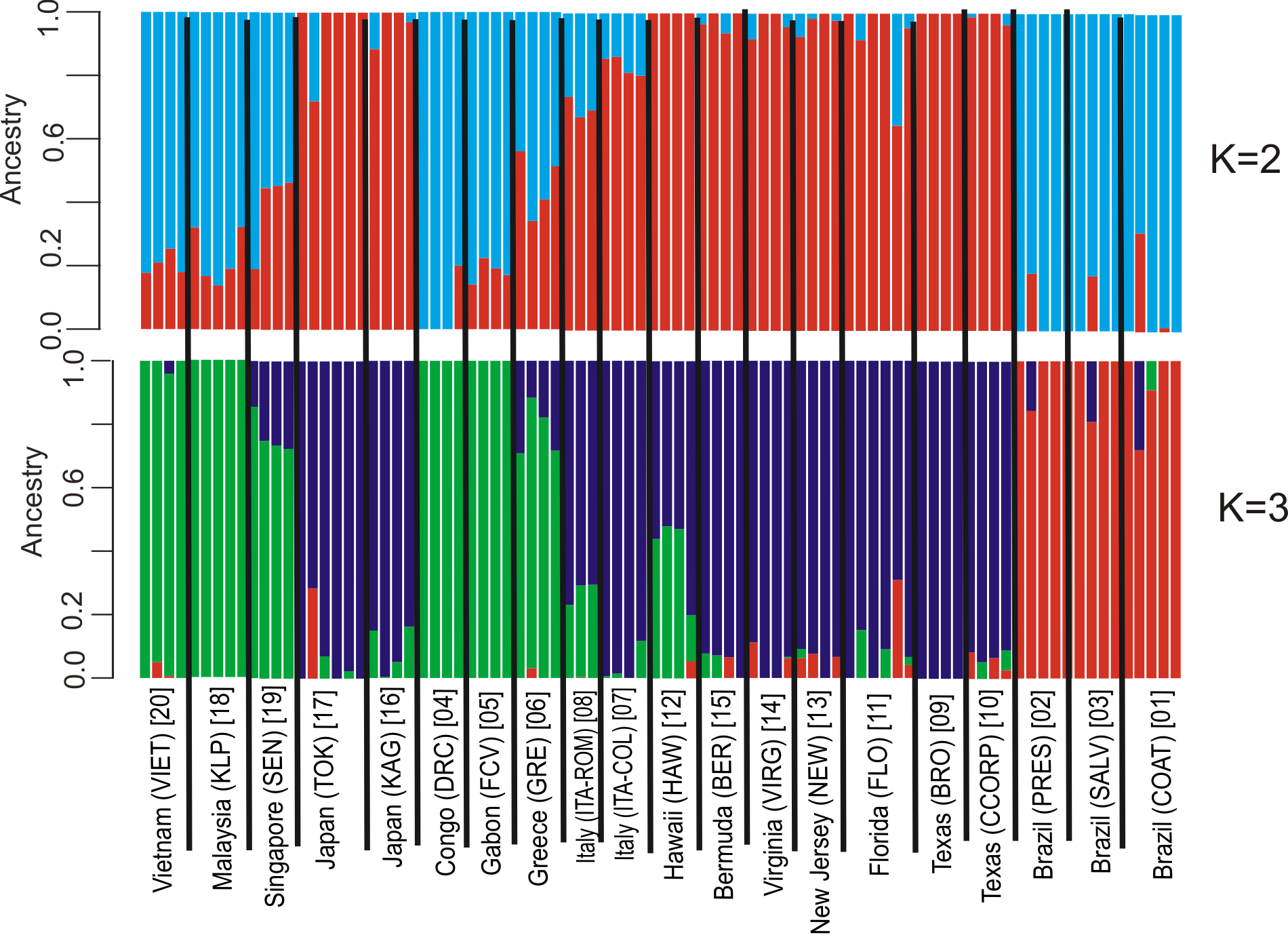


**Figure S4.** Admixture analysis on the reference genome assembly for the global dataset, retrieved with filtering for minimum mapping quality Q20.

**References**

Eaton DA (2014) PyRAD: assembly of de novo RADseq loci for phylogenetic analyses. *Bioinformatics* **30**, 1844-1849.

Excoffier L, Lischer HEL (2010) Arlequin suite ver 3.5: a new series of programs to perform population genetics analyses under Linux and Windows. *Molecular ecology resources* **10**, 564-567.

Razkin O, Sonet G, Breugelmans K*, et al.* (2016) Species limits, interspecific hybridization and phylogeny in the cryptic land snail complex Pyramidula: The power of RADseq data. *Molecular Phylogenetics and Evolution* **101**, 267-278.

Viricel A, Pante E, Dabin W, Simon-Bouhet B (2014) Applicability of RAD-tag genotyping for interfamilial comparisons: empirical data from two cetaceans. *Molecular ecology resources* **14**, 597-605.
